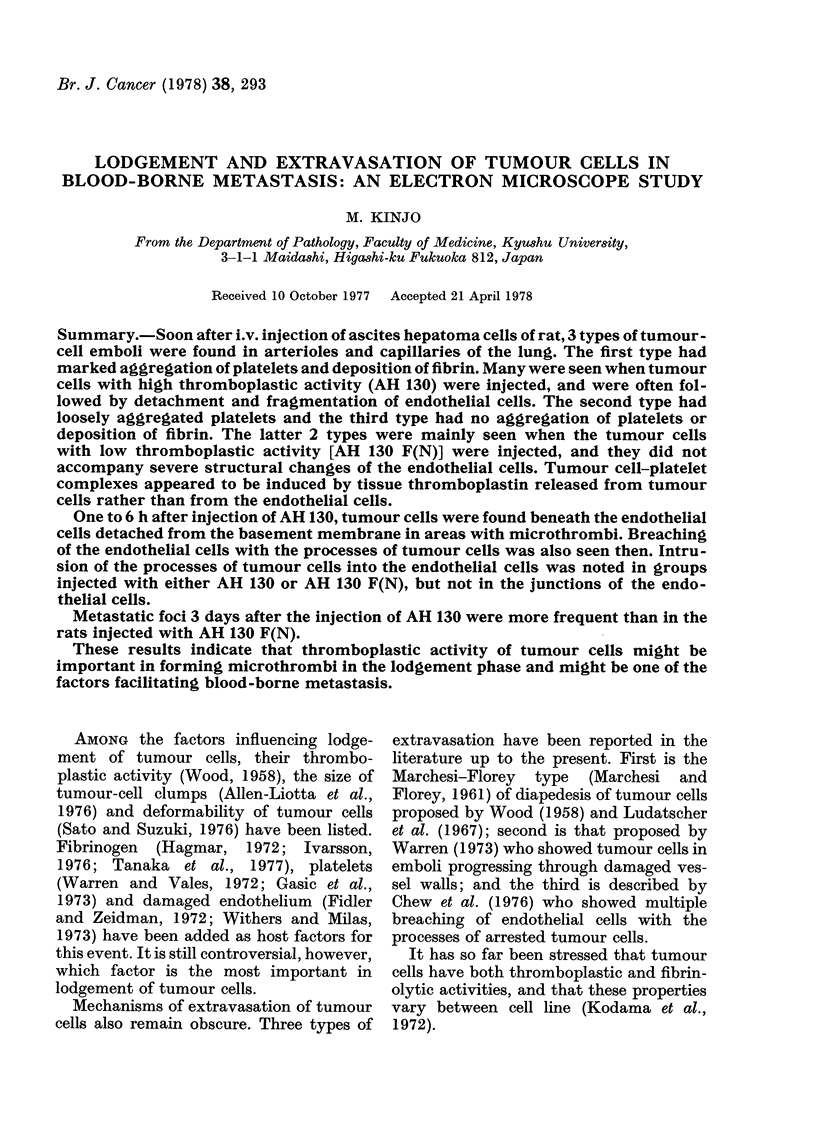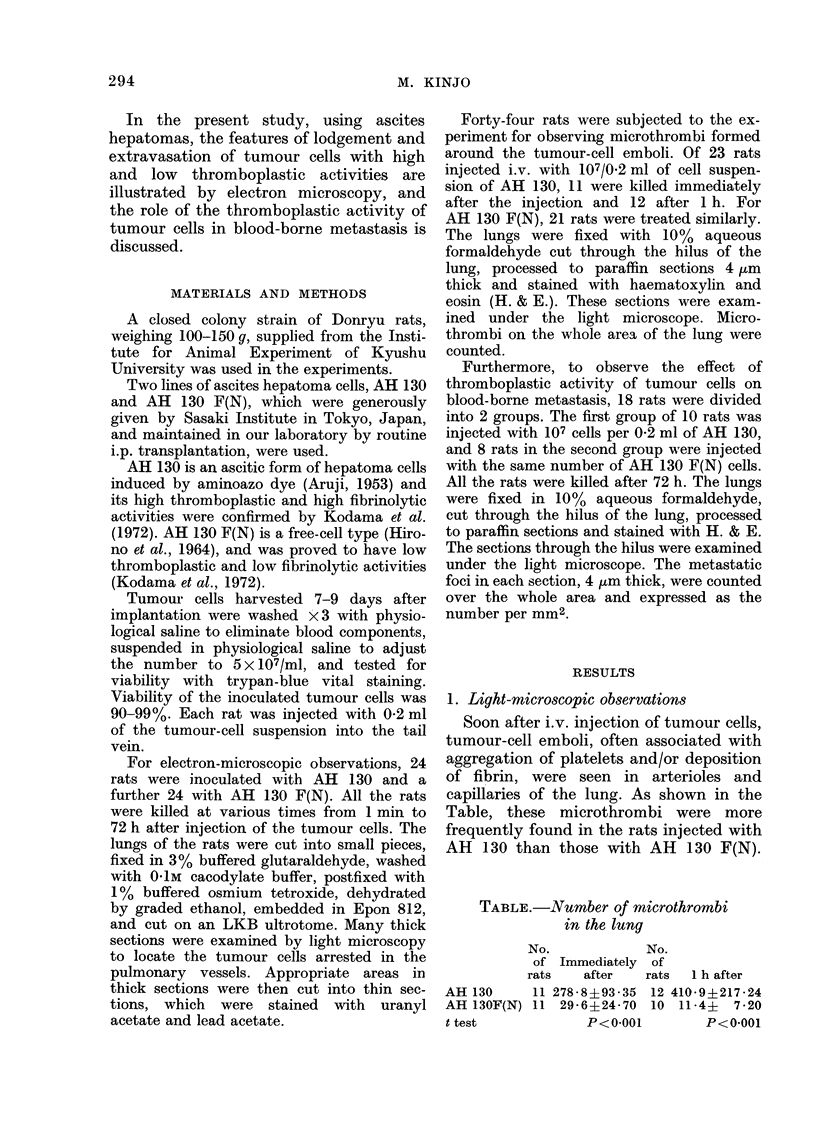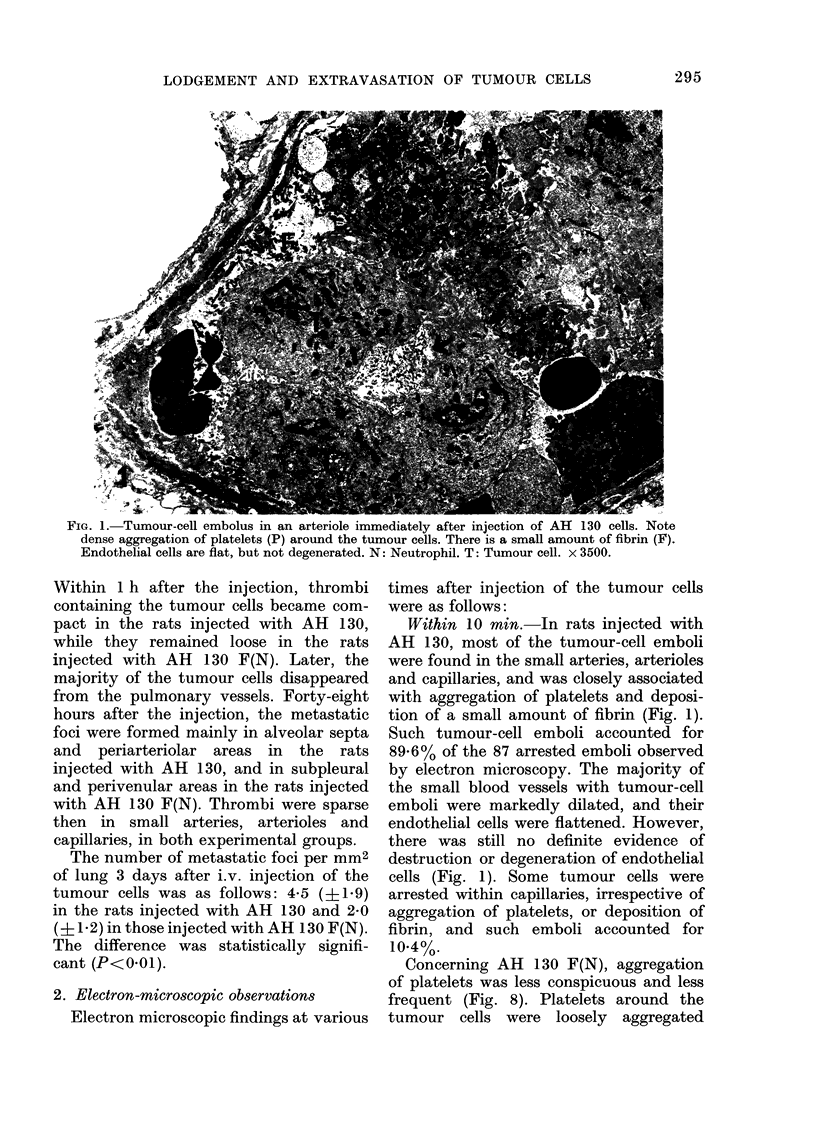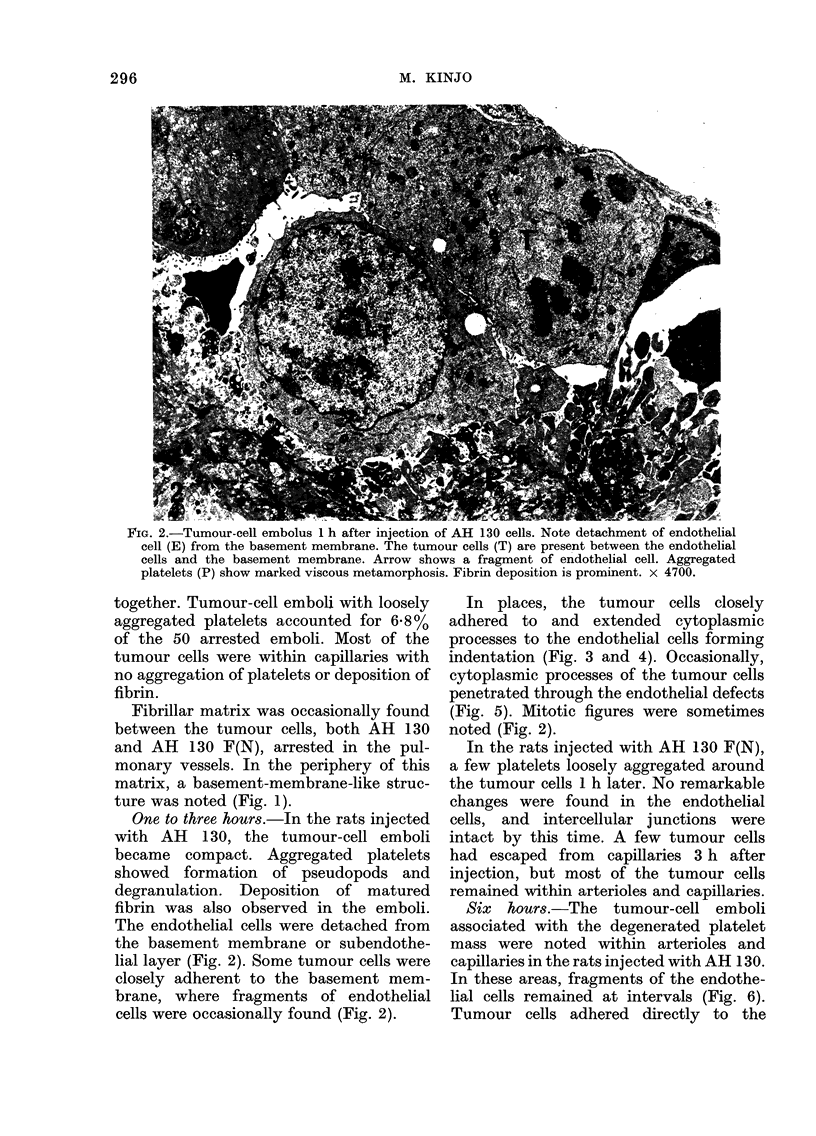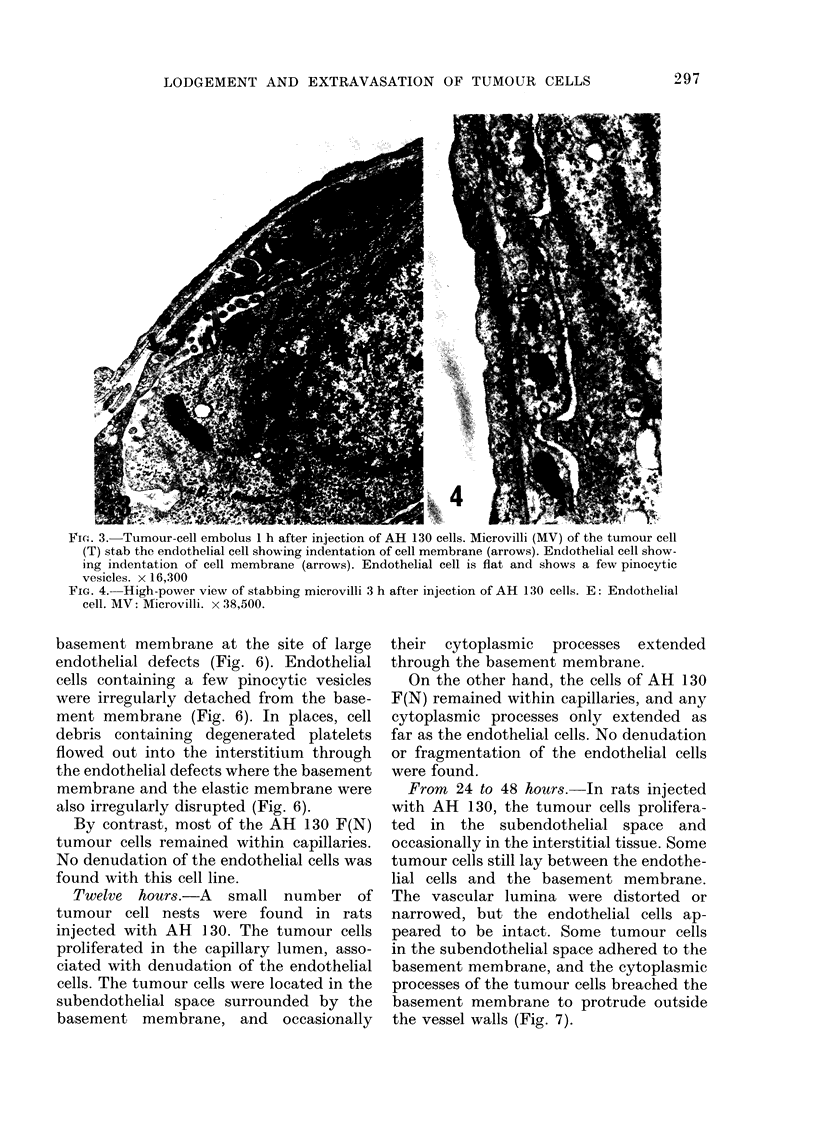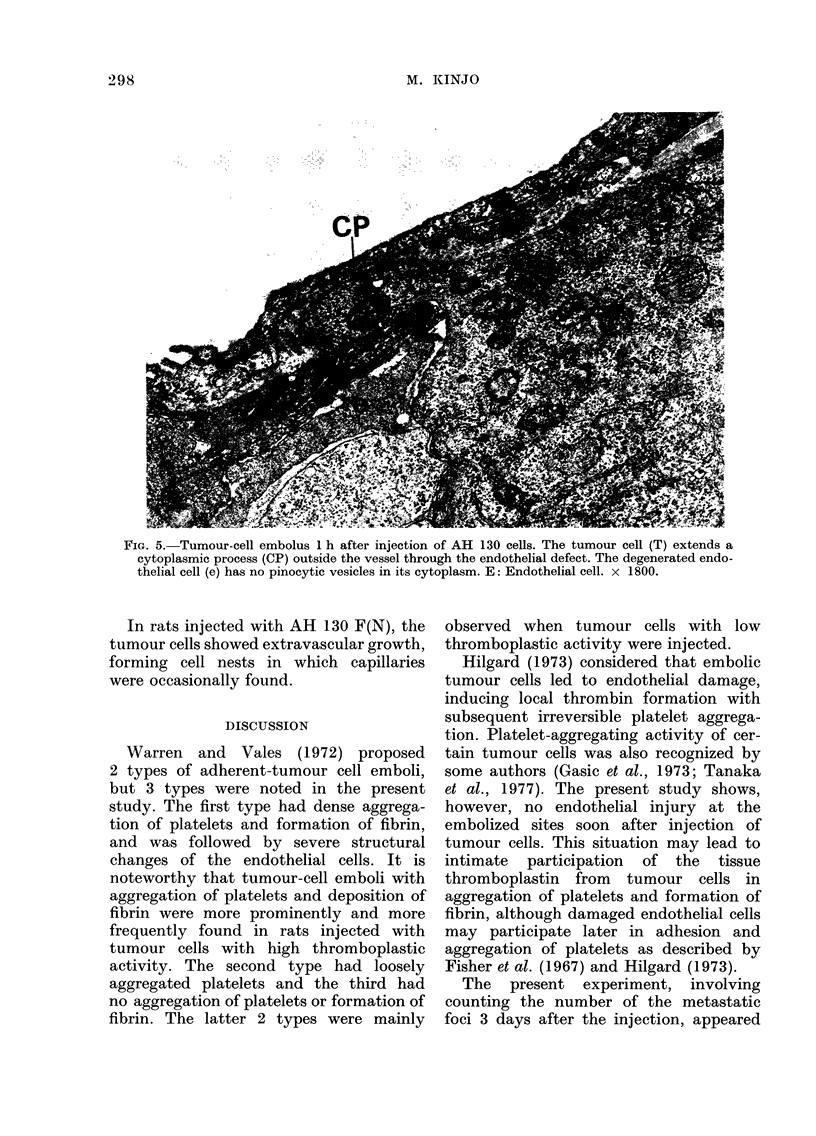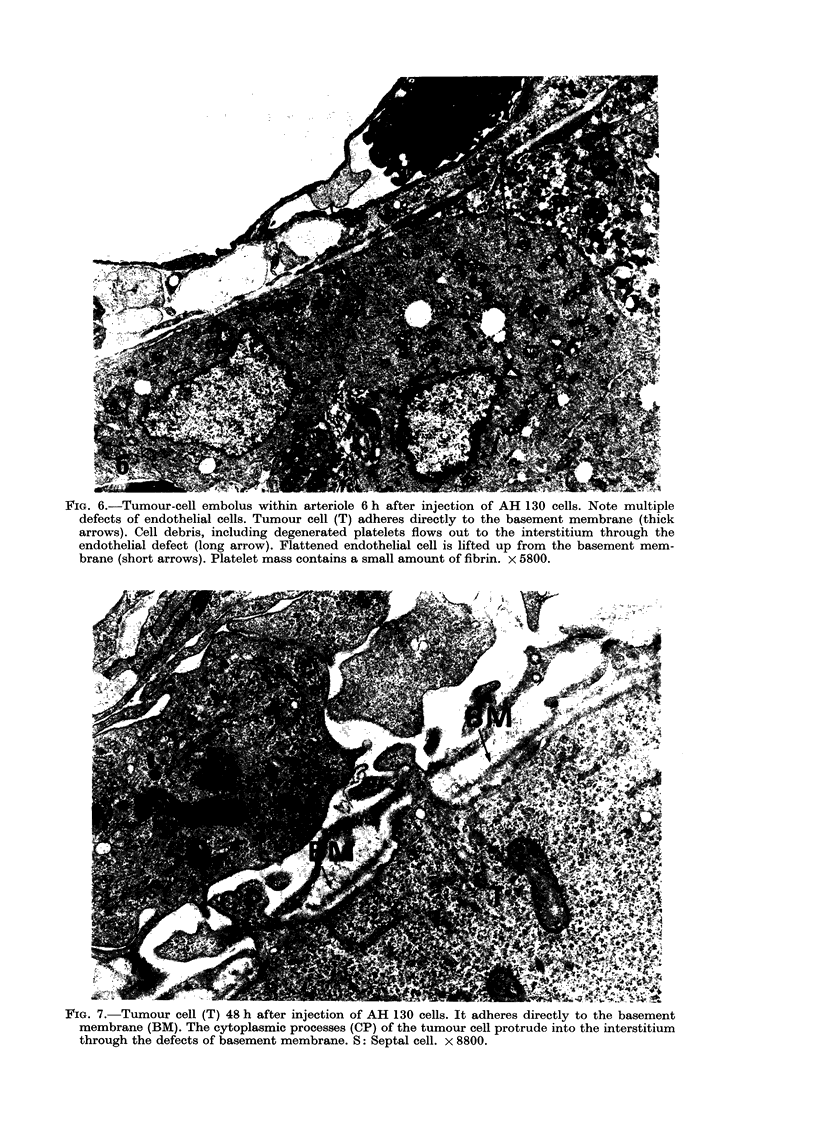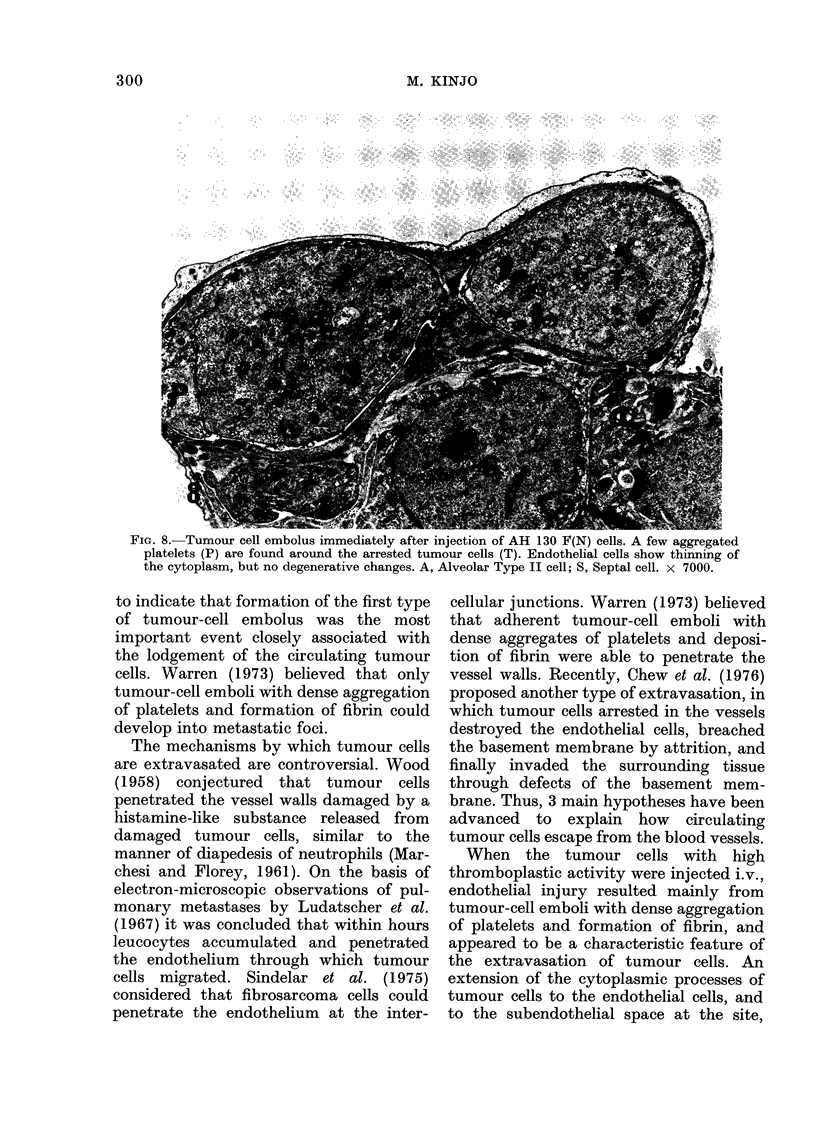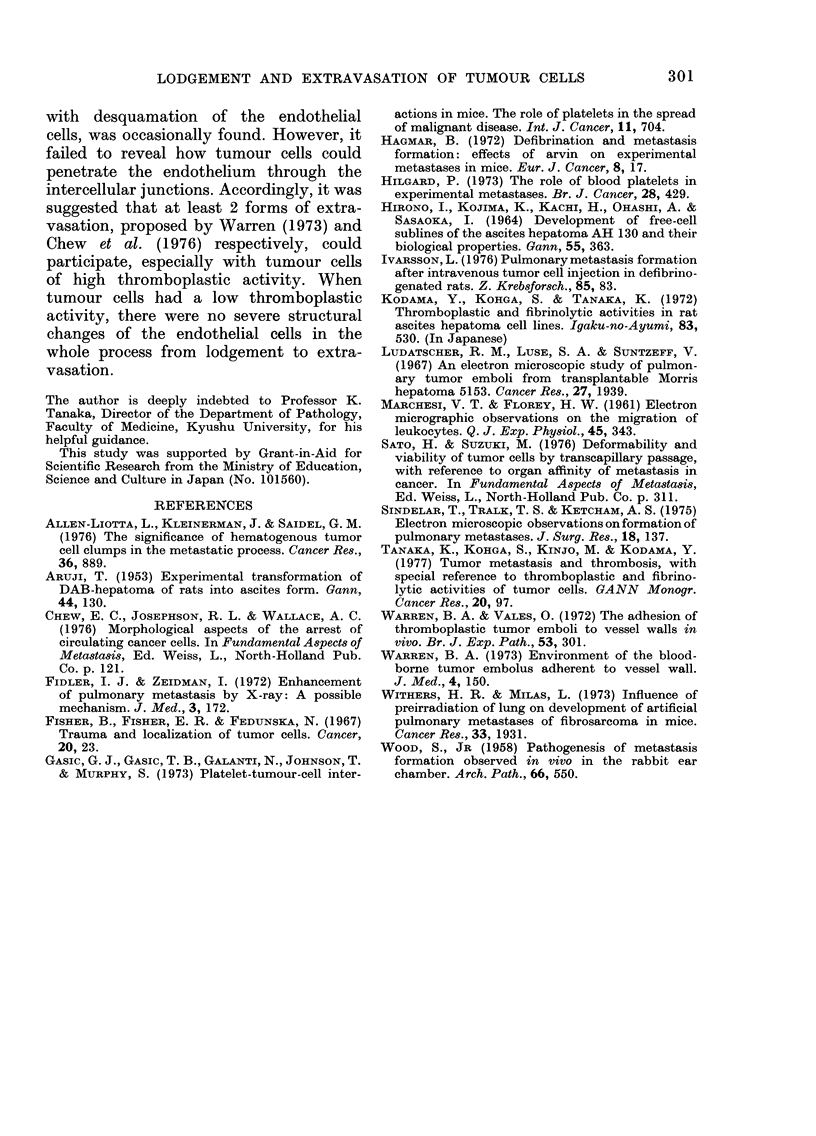# Lodgement and extravasation of tumour cells in blood-borne metastasis: an electron microscope study.

**DOI:** 10.1038/bjc.1978.201

**Published:** 1978-08

**Authors:** M. Kinjo

## Abstract

**Images:**


*Br. J. Cancer* (1978) **38**, 293

### LODGEMENT AND EXTRAVASATION OF TUMOUR CELLS IN BLOOD-BORNE METASTASIS: AN ELECTRON MICROSCOPE STUDY

M. KINJO

*From the Department of Pathology, Faculty of Medicine, Kyushu University, 3-1-1 Maidashi, Higashi-ku Fukuoka 812, Japan*

Received 10 October 1977 Accepted 21 April 1978

**Summary.—Soon after i.v. injection of ascites hepatoma cells of rat, 3 types of tumour-cell emboli were found in arterioles and capillaries of the lung. The first type had marked aggregation of platelets and deposition of fibrin. Many were seen when tumour cells with high thromboplastic activity (AH 130) were injected, and were often followed by detachment and fragmentation of endothelial cells. The second type had loosely aggregated platelets and the third type had no aggregation of platelets or deposition of fibrin. The latter 2 types were mainly seen when the tumour cells with low thromboplastic activity [AH 130 F(N)] were injected, and they did not accompany severe structural changes of the endothelial cells. Tumour cell–platelet complexes appeared to be induced by tissue thromboplastin released from tumour cells rather than from the endothelial cells.**

**One to 6 h after injection of AH 130, tumour cells were found beneath the endothelial cells detached from the basement membrane in areas with microthrombi. Breaching of the endothelial cells with the processes of tumour cells was also seen then. Intrusion of the processes of tumour cells into the endothelial cells was noted in groups injected with either AH 130 or AH 130 F(N), but not in the junctions of the endothelial cells.**

**Metastatic foci 3 days after the injection of AH 130 were more frequent than in the rats injected with AH 130 F(N).**

**These results indicate that thromboplastic activity of tumour cells might be important in forming microthrombi in the lodgement phase and might be one of the factors facilitating blood-borne metastasis.**

AMONG the factors influencing lodgement of tumour cells, their thromboplastic activity (Wood, 1958), the size of tumour-cell clumps (Allen-Liotta *et al.*, 1976) and deformability of tumour cells (Sato and Suzuki, 1976) have been listed. Fibrinogen (Hagmar, 1972; Ivarsson, 1976; Tanaka *et al.*, 1977), platelets (Warren and Vales, 1972; Gasic *et al.*, 1973) and damaged endothelium (Fidler and Zeidman, 1972; Withers and Milas, 1973) have been added as host factors for this event. It is still controversial, however, which factor is the most important in lodgement of tumour cells.

Mechanisms of extravasation of tumour cells also remain obscure. Three types of extravasation have been reported in the literature up to the present. First is the Marchesi–Florey type (Marchesi and Florey, 1961) of diapedesis of tumour cells proposed by Wood (1958) and Ludatscher *et al.* (1967); second is that proposed by Warren (1973) who showed tumour cells in emboli progressing through damaged vessel walls; and the third is described by Chew *et al.* (1976) who showed multiple breaching of endothelial cells with the processes of arrested tumour cells.

It has so far been stressed that tumour cells have both thromboplastic and fibrinolytic activities, and that these properties vary between cell line (Kodama *et al.*, 1972).

In the present study, using ascites hepatomas, the features of lodgement and extravasation of tumour cells with high and low thromboplastic activities are illustrated by electron microscopy, and the role of the thromboplastic activity of tumour cells in blood-borne metastasis is discussed.

### MATERIALS AND METHODS

A closed colony strain of Donryu rats, weighing 100–150 *g*, supplied from the Institute for Animal Experiment of Kyushu University was used in the experiments.

Two lines of ascites hepatoma cells, AH 130 and AH 130 F(N), which were generously given by Sasaki Institute in Tokyo, Japan, and maintained in our laboratory by routine i.p. transplantation, were used.

AH 130 is an ascitic form of hepatoma cells induced by aminoazo dye (Aruji, 1953) and its high thromboplastic and high fibrinolytic activities were confirmed by Kodama *et al.* (1972). AH 130 F(N) is a free-cell type (Hirono *et al.*, 1964), and was proved to have low thromboplastic and low fibrinolytic activities (Kodama *et al.*, 1972).

Tumour cells harvested 7–9 days after implantation were washed $\times 3$ with physiological saline to eliminate blood components, suspended in physiological saline to adjust the number to $5 \times 10^7$/ml, and tested for viability with trypan-blue vital staining. Viability of the inoculated tumour cells was 90–99%. Each rat was injected with 0·2 ml of the tumour-cell suspension into the tail vein.

For electron-microscopic observations, 24 rats were inoculated with AH 130 and a further 24 with AH 130 F(N). All the rats were killed at various times from 1 min to 72 h after injection of the tumour cells. The lungs of the rats were cut into small pieces, fixed in 3% buffered glutaraldehyde, washed with 0·1M cacodylate buffer, postfixed with 1% buffered osmium tetroxide, dehydrated by graded ethanol, embedded in Epon 812, and cut on an LKB ultrotome. Many thick sections were examined by light microscopy to locate the tumour cells arrested in the pulmonary vessels. Appropriate areas in thick sections were then cut into thin sections, which were stained with uranyl acetate and lead acetate.

Forty-four rats were subjected to the experiment for observing microthrombi formed around the tumour-cell emboli. Of 23 rats injected i.v. with $10^7$/0·2 ml of cell suspension of AH 130, 11 were killed immediately after the injection and 12 after 1 h. For AH 130 F(N), 21 rats were treated similarly. The lungs were fixed with 10% aqueous formaldehyde cut through the hilus of the lung, processed to paraffin sections 4 $\mu$m thick and stained with haematoxylin and eosin (H. & E.). These sections were examined under the light microscope. Microthrombi on the whole area of the lung were counted.

Furthermore, to observe the effect of thromboplastic activity of tumour cells on blood-borne metastasis, 18 rats were divided into 2 groups. The first group of 10 rats was injected with $10^7$ cells per 0·2 ml of AH 130, and 8 rats in the second group were injected with the same number of AH 130 F(N) cells. All the rats were killed after 72 h. The lungs were fixed in 10% aqueous formaldehyde, cut through the hilus of the lung, processed to paraffin sections and stained with H. & E. The sections through the hilus were examined under the light microscope. The metastatic foci in each section, 4 $\mu$m thick, were counted over the whole area and expressed as the number per mm$^2$.

### RESULTS

### 1. *Light-microscopic observations*

Soon after i.v. injection of tumour cells, tumour-cell emboli, often associated with aggregation of platelets and/or deposition of fibrin, were seen in arterioles and capillaries of the lung. As shown in the Table, these microthrombi were more frequently found in the rats injected with AH 130 than those with AH 130 F(N).

TABLE.—*Number of microthrombi in the lung*

| | No. of rats | Immediately after | No. of rats | 1 h after |
|---|---|---|---|---|
| AH 130 | 11 | 278·8±93·35 | 12 | 410·9±217·24 |
| AH 130F(N) | 11 | 29·6±24·70 | 10 | 11·4± 7·20 |
| *t* test | | $P<0.001$ | | $P<0.001$ |

FIG. 1.—Tumour-cell embolus in an arteriole immediately after injection of AH 130 cells. Note dense aggregation of platelets (P) around the tumour cells. There is a small amount of fibrin (F). Endothelial cells are flat, but not degenerated. N: Neutrophil. T: Tumour cell. ×3500.

Within 1 h after the injection, thrombi containing the tumour cells became compact in the rats injected with AH 130, while they remained loose in the rats injected with AH 130 F(N). Later, the majority of the tumour cells disappeared from the pulmonary vessels. Forty-eight hours after the injection, the metastatic foci were formed mainly in alveolar septa and periarteriolar areas in the rats injected with AH 130, and in subpleural and perivenular areas in the rats injected with AH 130 F(N). Thrombi were sparse then in small arteries, arterioles and capillaries, in both experimental groups.

The number of metastatic foci per $mm^2$ of lung 3 days after i.v. injection of the tumour cells was as follows: 4·5 (±1·9) in the rats injected with AH 130 and 2·0 (±1·2) in those injected with AH 130 F(N). The difference was statistically significant ($P<0·01$).

2. *Electron-microscopic observations*

Electron microscopic findings at various times after injection of the tumour cells were as follows:

*Within* 10 *min.*—In rats injected with AH 130, most of the tumour-cell emboli were found in the small arteries, arterioles and capillaries, and was closely associated with aggregation of platelets and deposition of a small amount of fibrin (Fig. 1). Such tumour-cell emboli accounted for 89·6% of the 87 arrested emboli observed by electron microscopy. The majority of the small blood vessels with tumour-cell emboli were markedly dilated, and their endothelial cells were flattened. However, there was still no definite evidence of destruction or degeneration of endothelial cells (Fig. 1). Some tumour cells were arrested within capillaries, irrespective of aggregation of platelets, or deposition of fibrin, and such emboli accounted for 10·4%.

Concerning AH 130 F(N), aggregation of platelets was less conspicuous and less frequent (Fig. 8). Platelets around the tumour cells were loosely aggregated

FIG. 2.—Tumour-cell embolus 1 h after injection of AH 130 cells. Note detachment of endothelial cell (E) from the basement membrane. The tumour cells (T) are present between the endothelial cells and the basement membrane. Arrow shows a fragment of endothelial cell. Aggregated platelets (P) show marked viscous metamorphosis. Fibrin deposition is prominent. × 4700.

together. Tumour-cell emboli with loosely aggregated platelets accounted for 6·8% of the 50 arrested emboli. Most of the tumour cells were within capillaries with no aggregation of platelets or deposition of fibrin.

Fibrillar matrix was occasionally found between the tumour cells, both AH 130 and AH 130 F(N), arrested in the pulmonary vessels. In the periphery of this matrix, a basement-membrane-like structure was noted (Fig. 1).

*One to three hours.*—In the rats injected with AH 130, the tumour-cell emboli became compact. Aggregated platelets showed formation of pseudopods and degranulation. Deposition of matured fibrin was also observed in the emboli. The endothelial cells were detached from the basement membrane or subendothelial layer (Fig. 2). Some tumour cells were closely adherent to the basement membrane, where fragments of endothelial cells were occasionally found (Fig. 2).

In places, the tumour cells closely adhered to and extended cytoplasmic processes to the endothelial cells forming indentation (Fig. 3 and 4). Occasionally, cytoplasmic processes of the tumour cells penetrated through the endothelial defects (Fig. 5). Mitotic figures were sometimes noted (Fig. 2).

In the rats injected with AH 130 F(N), a few platelets loosely aggregated around the tumour cells 1 h later. No remarkable changes were found in the endothelial cells, and intercellular junctions were intact by this time. A few tumour cells had escaped from capillaries 3 h after injection, but most of the tumour cells remained within arterioles and capillaries.

*Six hours.*—The tumour-cell emboli associated with the degenerated platelet mass were noted within arterioles and capillaries in the rats injected with AH 130. In these areas, fragments of the endothelial cells remained at intervals (Fig. 6). Tumour cells adhered directly to the

FIG. 3.—Tumour-cell embolus 1 h after injection of AH 130 cells. Microvilli (MV) of the tumour cell (T) stab the endothelial cell showing indentation of cell membrane (arrows). Endothelial cell showing indentation of cell membrane (arrows). Endothelial cell is flat and shows a few pinocytic vesicles. ×16,300

FIG. 4.—High-power view of stabbing microvilli 3 h after injection of AH 130 cells. E: Endothelial cell. MV: Microvilli. ×38,500.

basement membrane at the site of large endothelial defects (Fig. 6). Endothelial cells containing a few pinocytic vesicles were irregularly detached from the basement membrane (Fig. 6). In places, cell debris containing degenerated platelets flowed out into the interstitium through the endothelial defects where the basement membrane and the elastic membrane were also irregularly disrupted (Fig. 6).

By contrast, most of the AH 130 F(N) tumour cells remained within capillaries. No denudation of the endothelial cells was found with this cell line.

*Twelve hours.*—A small number of tumour cell nests were found in rats injected with AH 130. The tumour cells proliferated in the capillary lumen, associated with denudation of the endothelial cells. The tumour cells were located in the subendothelial space surrounded by the basement membrane, and occasionally their cytoplasmic processes extended through the basement membrane.

On the other hand, the cells of AH 130 F(N) remained within capillaries, and any cytoplasmic processes only extended as far as the endothelial cells. No denudation or fragmentation of the endothelial cells were found.

*From* 24 *to* 48 *hours.*—In rats injected with AH 130, the tumour cells proliferated in the subendothelial space and occasionally in the interstitial tissue. Some tumour cells still lay between the endothelial cells and the basement membrane. The vascular lumina were distorted or narrowed, but the endothelial cells appeared to be intact. Some tumour cells in the subendothelial space adhered to the basement membrane, and the cytoplasmic processes of the tumour cells breached the basement membrane to protrude outside the vessel walls (Fig. 7).

FIG. 5.—Tumour-cell embolus 1 h after injection of AH 130 cells. The tumour cell (T) extends a cytoplasmic process (CP) outside the vessel through the endothelial defect. The degenerated endothelial cell (e) has no pinocytic vesicles in its cytoplasm. E: Endothelial cell. × 1800.

In rats injected with AH 130 F(N), the tumour cells showed extravascular growth, forming cell nests in which capillaries were occasionally found.

### DISCUSSION

Warren and Vales (1972) proposed 2 types of adherent-tumour cell emboli, but 3 types were noted in the present study. The first type had dense aggregation of platelets and formation of fibrin, and was followed by severe structural changes of the endothelial cells. It is noteworthy that tumour-cell emboli with aggregation of platelets and deposition of fibrin were more prominently and more frequently found in rats injected with tumour cells with high thromboplastic activity. The second type had loosely aggregated platelets and the third had no aggregation of platelets or formation of fibrin. The latter 2 types were mainly observed when tumour cells with low thromboplastic activity were injected.

Hilgard (1973) considered that embolic tumour cells led to endothelial damage, inducing local thrombin formation with subsequent irreversible platelet aggregation. Platelet-aggregating activity of certain tumour cells was also recognized by some authors (Gasic *et al.*, 1973; Tanaka *et al.*, 1977). The present study shows, however, no endothelial injury at the embolized sites soon after injection of tumour cells. This situation may lead to intimate participation of the tissue thromboplastin from tumour cells in aggregation of platelets and formation of fibrin, although damaged endothelial cells may participate later in adhesion and aggregation of platelets as described by Fisher *et al.* (1967) and Hilgard (1973).

The present experiment, involving counting the number of the metastatic foci 3 days after the injection, appeared

FIG. 6.—Tumour-cell embolus within arteriole 6 h after injection of AH 130 cells. Note multiple defects of endothelial cells. Tumour cell (T) adheres directly to the basement membrane (thick arrows). Cell debris, including degenerated platelets flows out to the interstitium through the endothelial defect (long arrow). Flattened endothelial cell is lifted up from the basement membrane (short arrows). Platelet mass contains a small amount of fibrin. ×5800.

FIG. 7.—Tumour cell (T) 48 h after injection of AH 130 cells. It adheres directly to the basement membrane (BM). The cytoplasmic processes (CP) of the tumour cell protrude into the interstitium through the defects of basement membrane. S: Septal cell. ×8800.

FIG. 8.—Tumour cell embolus immediately after injection of AH 130 F(N) cells. A few aggregated platelets (P) are found around the arrested tumour cells (T). Endothelial cells show thinning of the cytoplasm, but no degenerative changes. A, Alveolar Type II cell; S, Septal cell. × 7000.

to indicate that formation of the first type of tumour-cell embolus was the most important event closely associated with the lodgement of the circulating tumour cells. Warren (1973) believed that only tumour-cell emboli with dense aggregation of platelets and formation of fibrin could develop into metastatic foci.

The mechanisms by which tumour cells are extravasated are controversial. Wood (1958) conjectured that tumour cells penetrated the vessel walls damaged by a histamine-like substance released from damaged tumour cells, similar to the manner of diapedesis of neutrophils (Marchesi and Florey, 1961). On the basis of electron-microscopic observations of pulmonary metastases by Ludatscher *et al.* (1967) it was concluded that within hours leucocytes accumulated and penetrated the endothelium through which tumour cells migrated. Sindelar *et al.* (1975) considered that fibrosarcoma cells could penetrate the endothelium at the intercellular junctions. Warren (1973) believed that adherent tumour-cell emboli with dense aggregates of platelets and deposition of fibrin were able to penetrate the vessel walls. Recently, Chew *et al.* (1976) proposed another type of extravasation, in which tumour cells arrested in the vessels destroyed the endothelial cells, breached the basement membrane by attrition, and finally invaded the surrounding tissue through defects of the basement membrane. Thus, 3 main hypotheses have been advanced to explain how circulating tumour cells escape from the blood vessels.

When the tumour cells with high thromboplastic activity were injected i.v., endothelial injury resulted mainly from tumour-cell emboli with dense aggregation of platelets and formation of fibrin, and appeared to be a characteristic feature of the extravasation of tumour cells. An extension of the cytoplasmic processes of tumour cells to the endothelial cells, and to the subendothelial space at the site, with desquamation of the endothelial cells, was occasionally found. However, it failed to reveal how tumour cells could penetrate the endothelium through the intercellular junctions. Accordingly, it was suggested that at least 2 forms of extravasation, proposed by Warren (1973) and Chew *et al.* (1976) respectively, could participate, especially with tumour cells of high thromboplastic activity. When tumour cells had a low thromboplastic activity, there were no severe structural changes of the endothelial cells in the whole process from lodgement to extravasation.

The author is deeply indebted to Professor K. Tanaka, Director of the Department of Pathology, Faculty of Medicine, Kyushu University, for his helpful guidance.

This study was supported by Grant-in-Aid for Scientific Research from the Ministry of Education, Science and Culture in Japan (No. 101560).

### REFERENCES

ALLEN-LIOTTA, L., KLEINERMAN, J. & SAIDEL, G. M. (1976) The significance of hematogenous tumor cell clumps in the metastatic process. *Cancer Res.*, **36**, 889.

ARUJI, T. (1953) Experimental transformation of DAB-hepatoma of rats into ascites form. *Gann*, **44**, 130.

CHEW, E. C., JOSEPHSON, R. L. & WALLACE, A. C. (1976) Morphological aspects of the arrest of circulating cancer cells. In *Fundamental Aspects of Metastasis*, Ed. Weiss, L., North-Holland Pub. Co. p. 121.

FIDLER, I. J. & ZEIDMAN, I. (1972) Enhancement of pulmonary metastasis by X-ray: A possible mechanism. *J. Med.*, **3**, 172.

FISHER, B., FISHER, E. R. & FEDUNSKA, N. (1967) Trauma and localization of tumor cells. *Cancer*, **20**, 23.

GASIC, G. J., GASIC, T. B., GALANTI, N., JOHNSON, T. & MURPHY, S. (1973) Platelet-tumour-cell interactions in mice. The role of platelets in the spread of malignant disease. *Int. J. Cancer*, **11**, 704.

HAGMAR, B. (1972) Defibrination and metastasis formation: effects of arvin on experimental metastases in mice. *Eur. J. Cancer*, **8**, 17.

HILGARD, P. (1973) The role of blood platelets in experimental metastases. *Br. J. Cancer*, **28**, 429.

HIRONO, I., KOJIMA, K., KACHI, H., OHASHI, A. & SASAOKA, I. (1964) Development of free-cell sublines of the ascites hepatoma AH 130 and their biological properties. *Gann*, **55**, 363.

IVARSSON, L. (1976) Pulmonary metastasis formation after intravenous tumor cell injection in defibrinogenated rats. *Z. Krebsforsch.*, **85**, 83.

KODAMA, Y., KOHGA, S. & TANAKA, K. (1972) Thromboplastic and fibrinolytic activities in rat ascites hepatoma cell lines. *Igaku-no-Ayumi*, **83**, 530. (In Japanese)

LUDATSCHER, R. M., LUSE, S. A. & SUNTZEFF, V. (1967) An electron microscopic study of pulmonary tumor emboli from transplantable Morris hepatoma 5153. *Cancer Res.*, **27**, 1939.

MARCHESI, V. T. & FLOREY, H. W. (1961) Electron micrographic observations on the migration of leukocytes. *Q. J. Exp. Physiol.*, **45**, 343.

SATO, H. & SUZUKI, M. (1976) Deformability and viability of tumor cells by transcapillary passage, with reference to organ affinity of metastasis in cancer. In *Fundamental Aspects of Metastasis*, Ed. Weiss, L., North-Holland Pub. Co. p. 311.

SINDELAR, T., TRALK, T. S. & KETCHAM, A. S. (1975) Electron microscopic observations on formation of pulmonary metastases. *J. Surg. Res.*, **18**, 137.

TANAKA, K., KOHGA, S., KINJO, M. & KODAMA, Y. (1977) Tumor metastasis and thrombosis, with special reference to thromboplastic and fibrinolytic activities of tumor cells. *GANN Monogr. Cancer Res.*, **20**, 97.

WARREN, B. A. & VALES, O. (1972) The adhesion of thromboplastic tumor emboli to vessel walls *in vivo*. *Br. J. Exp. Path.*, **53**, 301.

WARREN, B. A. (1973) Environment of the blood-borne tumor embolus adherent to vessel wall. *J. Med.*, **4**, 150.

WITHERS, H. R. & MILAS, L. (1973) Influence of preirradiation of lung on development of artificial pulmonary metastases of fibrosarcoma in mice. *Cancer Res.*, **33**, 1931.

WOOD, S., JR (1958) Pathogenesis of metastasis formation observed *in vivo* in the rabbit ear chamber. *Arch. Path.*, **66**, 550.